# Functional Properties and Potential Applications of Wheat Bran Extracts in Food and Cosmetics: A Review of Antioxidant, Enzyme-Inhibitory, and Anti-Aging Benefits

**DOI:** 10.3390/foods14030515

**Published:** 2025-02-05

**Authors:** Kaori Kobayashi, Md Suzauddula, Ryan Bender, Cheng Li, Yonghui Li, Xiuzhi Susan Sun, Weiqun Wang

**Affiliations:** 1 Department of Food Nutrition Dietetics and Health, Kansas State University, Manhattan, KS 66506, USA; kobakaori@ksu.edu (K.K.); suza@ksu.edu (M.S.); rdbender@ksu.edu (R.B.); 2 Department of Grain Science and Industry, Kansas State University, Manhattan, KS 66506, USA; chengli@ksu.edu; 3 Department of Biological and Agricultural Engineering, Kansas State University, Manhattan, KS 66506, USA; xss@ksu.edu

**Keywords:** wheat bran extracts, antioxidants, enzyme inhibition, anti-aging compounds, food application, cosmetic application, bioactive compounds, functional properties

## Abstract

This review examines existing studies on wheat bran extracts (WBEs) to provide an overview of their functional properties, including antioxidant and enzyme-inhibitory activities, highlighting their potential as natural alternatives for applications in both the food and cosmetic industries. Despite variations in extraction techniques, WBEs consistently demonstrated a significant presence of phenolic compounds and antioxidant activity. In the food industry, WBEs are valued for their nutritional richness, including dietary fiber, proteins, and bioactive compounds such as arabinoxylans. These compounds improve food texture, stability, and baking properties. Additionally, WBEs have demonstrated antimicrobial potential, enhanced product quality, and serve as natural preservatives. Furthermore, WBEs exhibit significant inhibitory effects against collagenase and elastase, suggesting promising anti-aging potential. In the cosmetics sector, WBEs have gained attention due to their emulsion stability, skin-whitening properties, antimicrobial effects, and antioxidant capacities. They have the potential to enhance the stability of cosmetic emulsions, improve skin hydration, and inhibit enzymes linked to skin aging, positioning WBEs as potentially natural alternatives to synthetic ingredients in skincare and anti-aging products. Our recent pilot study also supports that WBEs enhance antioxidant defenses against oxidative stress in rats, highlighting their potential role in anti-aging interventions. To further elucidate the efficacy and bioavailability of the beneficial bioactive compounds in WBEs for both food and cosmetic applications, more comprehensive in vivo studies are required in the future.

## 1. Introduction

Wheat bran contains a significant amount of bioactive compounds, particularly phenolic acids and flavonoids [1]. Wheat bran is the outermost layer of wheat grain, often considered a byproduct of the wheat milling industry. All over the world, approximately 0.12 billion tons of wheat bran is produced annually [2]. It is also an abundant and low-cost byproduct from agricultural processing, which can be used as an alternative food resource [3]. The antioxidant properties of wheat bran have also been documented in the scientific literature. These properties are primarily attributed to the presence of phenolic compounds, such as ferulic acid, p-coumaric acid, and vanillic acid [4]. Previous studies have conducted various in vitro assays to evaluate the antioxidant capacity of wheat bran extracts (WBEs), including DPPH (2,2-diphenyl-1-picrylhydrazyl) radical scavenging, ABTS (2,2’-casino-bis (3-ethylbenzothiazoline-6-sulfonic acid)) radical cation decolorization, and ferric-reducing antioxidant power (FRAP) assays, resulting in significant antioxidant activity of WBEs [5]. Furthermore, research has indicated that the antioxidant capacity of WBEs may vary depending on factors such as wheat variety, growing conditions, and extraction methods [6]. Antioxidants play a crucial role in human health by neutralizing harmful free radicals and reducing oxidative stress, which is implicated in various chronic diseases and the aging process [7]. These diseases represent a range of conditions affecting different body systems, from neurodegenerative disorders like Alzheimer’s and Parkinson’s to metabolic conditions like type 2 diabetes, as well as various forms of tissue degeneration seen in conditions like osteoarthritis and macular degeneration. In recent years, there has been a shift toward natural antioxidants due to concerns about the safety and efficacy of synthetic alternatives. Similarly, aging-related enzyme inhibitors have also gained prominence in both health and cosmetic industries [8]. On this point, WBEs can also be considered for such uses.

Wheat bran is also rich in dietary fibers, proteins, and fats, making it a valuable component in various industries, including food, feed, medicine, and fermentation [9,10]. Rich in dietary fiber, proteins, vitamins, and minerals, wheat bran has garnered significant attention in recent years because of its potential health benefits. Moreover, wheat bran contains various bioactive compounds, including phenolics and flavonoids, which contribute to its antioxidant properties [4]. As a result, there is growing interest in exploring the full potential of wheat bran as a functional food ingredient and a source of natural bioactive compounds for various applications. Wheat bran fiber plays a crucial role in digestive health by promoting regular bowel movements and preventing constipation [11]. The fermentation of wheat bran in the colon leads to the production of short-chain fatty acids, such as butyrate, which are beneficial for colon health [12]. The presence of butyrate-producing bacteria in the gut is enhanced by wheat bran consumption, which supports a healthy gut microbiome and may protect against colon cancer [12]. Regular consumption of whole grains, including wheat bran, is associated with a 20% to 40% reduction in the risk of cardiovascular diseases. This is due to the synergistic effects of the fiber and other constituents present in whole grains [13]. Wheat bran helps in reducing blood plasma cholesterol levels, which is a significant factor in preventing cardiovascular diseases [14].

The contamination and degradation of food products by microorganisms represent a significant challenge for the food industry, resulting in substantial detriment to economic systems and public health on a global scale [15]. Furthermore, losses incurred during storage have also been identified as a critical factor contributing to food waste [16]. Prolonging the shelf life of food products can markedly diminish waste by reducing the rates of spoilage and occurrences of stockouts, which is advantageous for both manufacturers and retailers. Additionally, an extended shelf life can facilitate cost reductions in production and distribution, as it promotes more efficient inventory management and mitigates the necessity of frequent replenishment [17]. Moreover, oxidation poses a significant issue within the food industry due to its profound effects on the quality, shelf life, and marketability of food and beverage products [18]. The food industry requires various ingredients, both synthetic and natural. Notably, numerous synthetic ingredients are experiencing declining popularity owing to health-related apprehensions, diminished nutritional values, and ongoing debates regarding their safety. In contrast, natural sweeteners tend to elicit positive consumer perceptions, as they are commonly associated with healthier lifestyles and enhanced nutritional values [19]. In order to assist food industries in mitigating oxidation and microbial threats, as well as extending shelf life, the identification of sustainable food ingredients and additives is of paramount importance. WBEs, which are abundant in natural antioxidants and bioactive compounds, emerge as a promising candidate in this endeavor. Utilizing such sustainable sources has the potential to improve product quality and stability while aligning with ecological and health-oriented objectives.

The cosmetic sector represents one of the most significant and rapidly expanding domains within the global economic framework, boasting an impressive revenue of USD 100.49 billion on an international scale [20]. The principal categories within the cosmetics market encompass skincare, haircare, makeup, perfumes, toiletries, and deodorants, as well as oral cosmetics, with skincare emerging as the predominant category in the year 2021 [21]. The incorporation of synthetic constituents in cosmetic formulations can precipitate a spectrum of adverse effects, predominantly attributable to the inclusion of allergenic and toxic agents. These adverse effects may manifest as mild dermal irritations or escalate to more grave health complications, including allergic responses and potential carcinogenic repercussions. The escalating prevalence of cosmetic usage globally has intensified apprehensions regarding these detrimental effects, thereby necessitating heightened awareness and regulatory oversight [22,23]. The literature indicates that a principal challenge confronting the cosmetic industry lies in the pursuit of safe and sustainable natural alternatives that can supplant synthetic materials utilized as ingredients in cosmetic formulations, owing to the associated environmental ramifications and the concomitant implications for human health, which are also of paramount concern to consumers. In this context, WBEs have exhibited considerable promise as a viable ingredient for the cosmetics industry due to their multifaceted functional properties. Investigations have demonstrated that WBEs can augment cosmetic formulations by imparting advantages such as skin whitening, antioxidation, wrinkle reduction, and hair care benefits [24]. WBEs are characterized by the presence of phenolic compounds and bioactive peptides that demonstrate substantial antioxidant capabilities. These compounds play a pivotal role in modulating cellular oxidative stress, a critical factor implicated in the aging process. Furthermore, the extracts have been evidenced to inhibit enzymes such as collagenase, elastase, and hyaluronidase, which are integral to the degradation of skin architecture and elasticity. This enzymatic inhibition suggests prospective applications in cosmetic products targeted at minimizing the appearance of skin wrinkles [25]. These properties could make WBEs a promising natural ingredient for various cosmetic applications.

Despite the extensive research on WBEs, several knowledge gaps persist. Firstly, there is limited information on its potential enzyme-inhibitory effects from animal studies, particularly against skin-related enzymes, such as collagenase, elastase, and hyaluronidase. Secondly, the relationship between the antioxidant properties of WBEs and their potential enzyme-inhibitory effects has not been thoroughly explored. Understanding this relationship could provide valuable insights into the mechanisms of action of wheat bran bioactive compounds. Thirdly, while various extraction methods have been used in previous studies, there is a lack of comprehensive comparisons between traditional solvent extraction and more novel techniques like subcritical water extraction, particularly in terms of their impact on both antioxidant and enzyme-inhibitory properties. Lastly, most studies have focused on in vitro assessments, leaving a gap in our understanding of the in vivo efficacy and bioavailability of wheat bran bioactive compounds. Addressing these knowledge gaps could significantly advance our understanding of wheat bran’s potential applications in the health, cosmetics, and food industries. In previous studies, the focus has primarily been on exploring the potential health benefits of wheat bran, specifically its antioxidant properties. However, our previous research uncovered new findings about the health potential of WBEs. These discoveries provide novel insights and indicate a more promising potential for health enhancement than has been reported to date.

## 2. Wheat Bran Extracts

### 2.1. Bioactive Compounds in Wheat Bran Extracts

**Figure 1:**
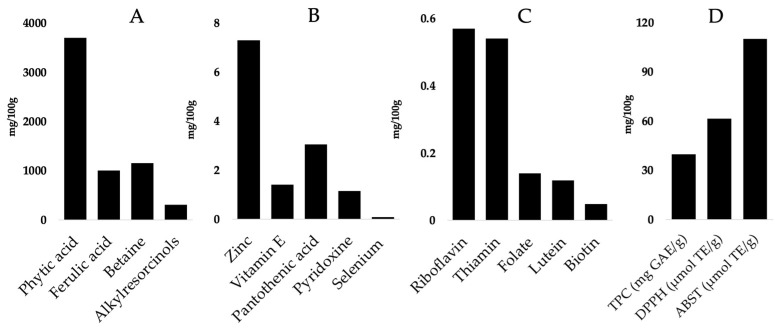
The contents of bioactive compounds, vitamins, minerals, and antioxidant activities of wheat bran extracts: (**A**) levels of phytochemicals; (**B**) mineral and vitamin contents; (**C**) additional vitamin levels; (**D**) antioxidant activities as measured by the total phenolic content (TPC) (expressed in mg gallic acid equivalents/g), DPPH radical scavenging activity (expressed in Trolox equivalence)/g), and ABTS radical scavenging activity (expressed in Trolox equivalence/g). Modified from [25,29].

Wheat bran is a rich source of bioactive compounds, which include phenolic acids, bioactive peptides, and dietary fibers, all contributing to its antioxidant and health-promoting properties (Figure 1). Significant amounts of phenolic acids are found in wheat bran, including ferulic acid, gallic acid, p-hydroxybenzoic acid, syringic acid, vanillic acid, and p-coumaric acid. These compounds are known for their antioxidant properties [26,27]. A study found that a wide range of bioactive compounds exists in wheat bran, namely, saponins (1.16 ± 0.10 mg diosgenin equivalents (DE)/g), gallic acid (16.57 ± 0.16–18.12 ± 2.01 mg/100 g), caffeic acid (0.86 ± 0.08–97.61 ± 9.60 mg/100 g), p-coumaric acid (19.68 ± 0.33–23.40 ± 0.64 mg/100 g), ferulic acid (9.06 ± 0.15–9.73 ± 0.09 mg/100 g), phytic acid (2180–5220 mg/100 g), alkylresorcinols (220–400 mg/100 g), vitamin E (1.4 mg/100 g), betaine (1000–1300 mg/100 g), choline (47–100 mg/100 g), niacin (14–18 mg/100 g), pantothenic acid (2.2–3.9 mg/100 g), riboflavin (0.39–0.75 mg/100 g), biotin (0.048 mg/100 g), thiamin (0.54 mg/100 g), pyridoxine (1–1.3 mg/100 g), folate (79–200 μg/100 g), lutein (97–140 μg/100 g), iron (11 mg/100 g), manganese (12 mg/100 g), zinc (7.3 mg/100 g), and selenium (78 μg/100 g) [28,29]. The existence of bioactive chemicals in wheat bran or WBEs indicates its attractive potential for industrial applications as food ingredients, nutraceuticals, and others [30]. Additionally, wheat bran contains significant amounts of flavonoids and carotenoids, which are known for their antioxidant properties. One study reported that wheat bran extracts contain flavonoids (185.96 ± 1.31 to 206.74 ± 4.80 µg catechin equivalents (CE)/g bran), anthocyanin (36.6 ± 0.10 to 63.0 ± 0.20 µg cyanidin 3-glucoside/g bran), beta carotene (6.11 ± 0.05 to 14.25 ± 0.12 µg/100 g bran), zeaxanthin (16.68 ± 0.23 to 35.21 ± 0.47 µg/100 g bran), and lutein (132.93 ± 0.82 to 174.59 ± 1.08 µg/100 g bran) [31]. Moreover, carotenoids and unsaturated fatty acids are also found in WBEs, contributing to their nutritional value and potential health benefits, such as reducing the risk of cardiovascular diseases [32,33]. Phytosterols are natural compounds found in plants that are similar in structure to cholesterol. Phytosterols are known for their cholesterol-lowering effects and may also help prevent obesity, diabetes, and cancer. Interestingly, phytosterols, particularly oxyphytosterols, have also been identified in wheat bran [34].

### 2.2. Bioactive Properties in Wheat Bran Extracts

Wheat bran is a significant source of dietary fibers, which are known for their role in cancer prevention, particularly colon and breast cancers. The fibers in wheat bran are complemented by various phytochemicals, including phytic acid, phenolic acids, lignans, and flavonoids, which also contribute to its protective effects against cancer [35,36]. A study investigated the anticancer effects of phytic acid. As a phytochemical, phytic acid demonstrates potent anti-cancer properties, particularly in colorectal cancer (CRC), through several mechanisms [37,38]. Phytic acid inhibits cell proliferation in CRC cell lines (HCT116 and HT-29) in a dose- and time-dependent manner, with IC50 values of 2.96 mm and 3.35 mm, respectively [38]. Phytic acid also induces mitochondrial intrinsic apoptosis via the caspase-9 and caspase-3 cascade, leading to cancer cell death [38]. Additionally, it causes cell cycle arrest at the G2/M phase, preventing further division and proliferation of cancer cells. Phytic acid reduces the expression of pro-inflammatory markers such as cyclooxygenase 2 (COX-2), inducible nitric oxide synthase (iNOS), interleukin-1 beta (IL-1β), interleukin-6 (IL-6), and interleukin-10 (IL-10), creating a less favorable environment for tumor growth [38]. Furthermore, phytic acid suppresses the nuclear factor kappa-light-chain-enhancer of activated B cells (NF-κB) and β-catenin signaling pathways, decreasing the expression of downstream targets like cyclin D1 and c-Myc, which are involved in cell proliferation and survival [38]. Together, these actions underscore phytic acid’s multifaceted role in cancer prevention.

The presence of ferulic acid, syringic acid, p-hydroxybenzoic acid, vanillic acid, and coumaric acid in wheat bran further enhances its antioxidant properties, which are crucial in preventing oxidative-stress-related diseases [39]. WBEs exhibit significant antioxidant activity, as evidenced by their high total phenolic and flavonoid contents [4]. A recent in vitro study demonstrated up to 61.36 μmol TE/g DPPH radical scavenging activity and 110.95 μmol TE/g ABTS+ scavenging activity (Figure 1D) in WBEs [25]. These antioxidants are effective in scavenging free radicals, such as DPPH and ABTS, and in reducing lipid peroxidation in human LDL, which is vital for cardiovascular health [39,40]. An in vivo study showed that ferulic acid operates through several biological mechanisms, primarily influencing hepatic functions by modulating specific signaling pathways [41]. Ferulic acid exerts protective effects on liver health by modulating hepatic functions through the inhibition of protein tyrosine phosphatase 1B (PTP1B) and the subsequent activation of adenosine monophosphate-activated protein kinase (AMPK) [41]. Ferulic acid directly binds to PTP1B, preventing its dephosphorylation of AMPK, a crucial regulator of energy homeostasis and cellular stress responses [41]. By activating AMPK, FA alleviates oxidative stress and inflammation, key contributors to liver fibrosis, and reduces the activation of hepatic stellate cells (HSCs), thereby decreasing pro-fibrotic factors. This cascade of events helps protect the liver from injury, fibrosis, and inflammation, offering a potential therapeutic approach for managing liver fibrosis and related metabolic disorders [41].

**Figure 2:**
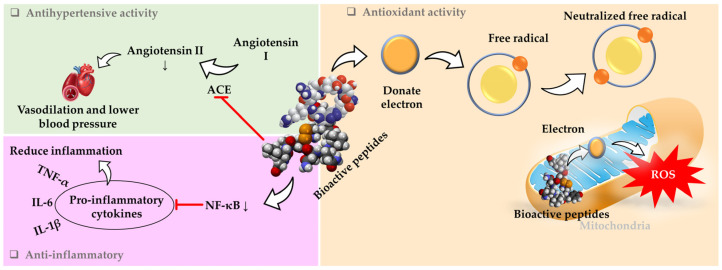
Potential mechanisms of the antioxidant, antihypertensive, and anti-inflammatory activities of wheat bran extracts and their bioactive peptides against reactive oxygen species. The top-left side of the image illustrates how hydrophobic amino acids contribute to the formation of peptides with antioxidant properties in the cytoplasm. The right side of the image depicts the role of hydrophobic amino acids in the formation of antioxidant peptides that neutralize reactive oxygen species (ROS) during cellular respiration by donating electrons. The bottom-left side shows the anti-inflammatory activity. The downward arrow (↓) indicates decreased levels of NF-κB or Angiotensin II.

Moreover, bioactive peptides derived from WBEs exhibit a range of physiological activities, including antioxidant, antihypertensive, and anti-inflammatory effects (Figure 2). These peptides are generated through enzymatic hydrolysis and have been identified for their potential health benefits. Generally, the peptides are characterized by their specific amino acid sequences, which contribute to their bioactivity and potential applications in nutraceuticals and functional foods. Peptides derived from wheat bran have shown significant antioxidant activities, particularly against oxidative stress in HepG2 cells.

The peptide sequences aspartic acid–leucine–aspartic acid–tryptophan (DLDW) and aspartic acid–leucine–glycine–leucine (DLGL), which have been identified in wheat bran extract, act as competitive inhibitors by binding to the Kelch domain of Keap1, specifically targeting the ETGE motif binding site [42]. This binding disrupts the natural interaction between Keap1 and Nrf2, reducing Nrf2 ubiquitination and preventing its degradation [42]. By stabilizing Nrf2, these peptides enhance the activation of antioxidant defense pathways, which are crucial for cellular protection [42]. DLDW and DLGL were identified for their high binding affinities, and they interfere with the Keap1–Nrf2 interaction—a critical pathway in antioxidant regulation [43]. Additionally, these peptides exhibit multifunctional bioactive properties. They act as competitive inhibitors of dipeptidyl peptidase IV (DPP-IV), an enzyme involved in glucose metabolism through the degradation of incretin hormones [44]. Furthermore, they show moderate inhibitory activity against angiotensin-converting enzyme (ACE), a key enzyme in blood pressure regulation, highlighting their potential in cardiovascular health and antihypertensive therapies [45].

Seven peptides, including NL, QL, FL, HAL, AAVL, AKTVF, and TPLTR, were identified from wheat bran protein hydrolysate. These peptides exhibited significant antihypertensive effects, with the <1 kDa fraction showing better absorption and interaction with ACE and renin, leading to decreased blood pressure in spontaneously hypertensive rats [46].

In addition, a specific wheat bran extract exhibited strong anti-inflammatory properties by reducing the expression of inflammatory markers such as iNOS and COX-2 in stimulated cells [5].

The anti-inflammatory properties of wheat bran extract have been investigated through various in vitro and in vivo studies. In vitro research has demonstrated that diferulic acid, derived from ferulic acid in wheat bran extract, exhibits significant anti-inflammatory activity through multiple mechanisms. A study showed that wheat bran hydrolysates containing diferulic acid effectively reduced pro-inflammatory cytokine secretion, specifically monocyte chemoattractant protein-1 (MCP-1) and tumor necrosis factor-alpha (TNF-α) in macrophages [47]. The study also revealed a synergistic effect when diferulic acids were combined with other bioactive compounds present in wheat bran, such as ferulic acid and various phenolic compounds, leading to enhanced anti-inflammatory effects [47]. Additional in vitro studies have demonstrated that bioactive peptides from wheat bran operate through the NF-κB signaling pathway, a crucial mediator of inflammatory responses. These peptides effectively inhibit this pathway, resulting in reduced expression of various pro-inflammatory cytokines, including TNF-α, IL-6, and IL-1β [48,49]. In vivo studies have provided further evidence of anti-inflammatory effects. In an animal model study, bioactive peptides demonstrated significant anti-inflammatory activity by reducing TNF-α and IL-6 concentrations in rat aqueous humor by 77.26% and 85.67%, respectively [48].

Additionally, a study indicated that the higher content of hydrophobic amino acids in wheat bran protein implies a potential for heightened antioxidant efficacy. Hydrophobic amino acids, such as alanine, valine, leucine, proline, methionine, phenylalanine, tryptophan, and isoleucine, have non-polar side chains that tend to avoid water. This property allows them to interact more effectively with lipid membranes and other hydrophobic environments, which is crucial for antioxidant activity (Figure 2). The greater presence of these amino acids could pose great potential for interaction with free radicals, which are often found in lipid-rich environments. These compounds are known to be major contributors to the antioxidant activity of plant extracts, and their quantification provides valuable insights into the composition and potential bioactivity of the extracts [50].

### 2.3. Anti-Aging Potential of Wheat Bran Extracts

**Figure 3:**
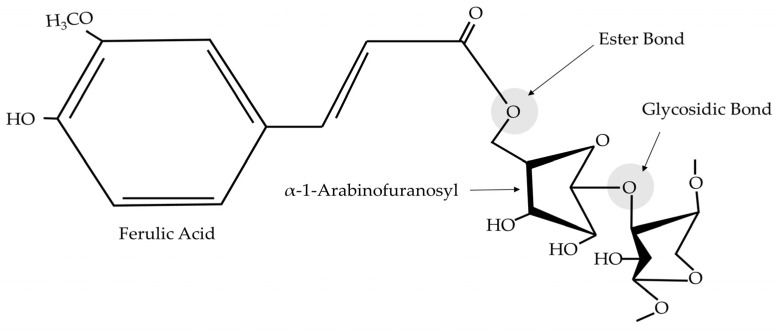
Chemical structures of arabinoxylans. Monosubstituted β-d-xylopyranosylat *O*-3 with ferulic acid residue esterified at α-l-arabinofuranosyl.

Aging is a complex biological process characterized by the gradual deterioration of physiological functions, leading to increased vulnerability to diseases and, ultimately, death. By retarding the aging process, we can avoid aging-related diseases, including cardiovascular diseases, cancer, and neurodegenerative disorders, and improve our quality of life. Antioxidants from wheat bran have potential anti-aging properties. Wheat bran contains a variety of phenolic compounds, including ferulic acid, which has shown strong antioxidant activities. These compounds can neutralize free radicals, which are implicated in cellular aging and oxidative stress-related diseases [51]. Recent studies have shown that WBEs can inhibit enzymes such as collagenase and elastase, which are involved in the breakdown of the skin’s structural proteins. This inhibition could potentially slow down skin-aging processes [4]. Chronic inflammation is associated with accelerated aging. Some components of wheat bran, particularly arabinoxylan, have shown anti-inflammatory properties, which could contribute to its anti-aging effects [52]. Arabinoxylans consist of a linear chain backbone of β-d-xylopyranosyl residues linked through glycosidic linkages (Figure 3). The combination of various bioactive compounds in wheat bran may provide synergistic effects, enhancing its overall anti-aging potential beyond what individual compounds might achieve [4].

**Figure 4:**
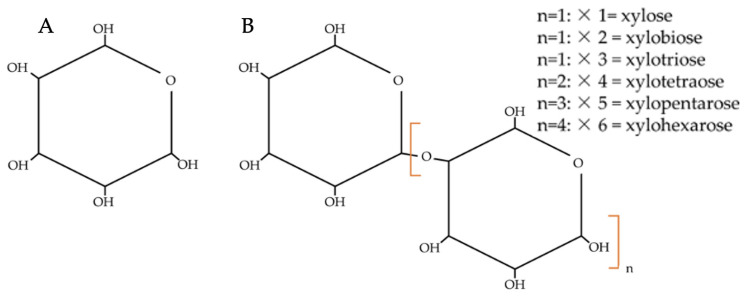
Chemical structures of (**A**) xylose and (**B**) xylo-oligosaccharides; “*n*” is a variable number of xylose units. The brackets ‘[ ]’ enclose a section of the molecular structure, showing that the portion repeats multiple times in the polymer chain.

The newly discovered antioxidants in WBEs, particularly xylo-oligosaccharides (XOS) (Figure 4) and protein hydrolysates, work as anti-aging agents by targeting oxidative stress and inhibiting enzymes related to skin aging, such as collagenase, elastase, and hyaluronidase (Figure 3 and Figure 4) [25]. Collagenase, elastase, and hyaluronidase enzymes are responsible for breaking down essential skin components, leading to wrinkles and loss of elasticity. By inhibiting these enzymes, the antioxidants from wheat bran or WBEs can maintain the skin’s structure and function. In addition, the phenolic compounds present in wheat bran have strong antioxidant properties, neutralizing free radicals and reducing oxidative damage at the cellular level. This reduction in oxidative stress plays a key role in slowing down the aging process and preventing age-related diseases. Specifically, the study shows that XOS from hard wheat bran has the highest hyaluronidase inhibition activity, reaching 49.9% at a 0.5 mg/mL concentration. These properties make WBEs promising candidates for cosmetic and therapeutic applications aimed at preventing aging and maintaining skin health.

### 2.4. Inhibitory Effect of Wheat Bran Extract on Skin Aging-Related Enzyme

**Figure 5:**
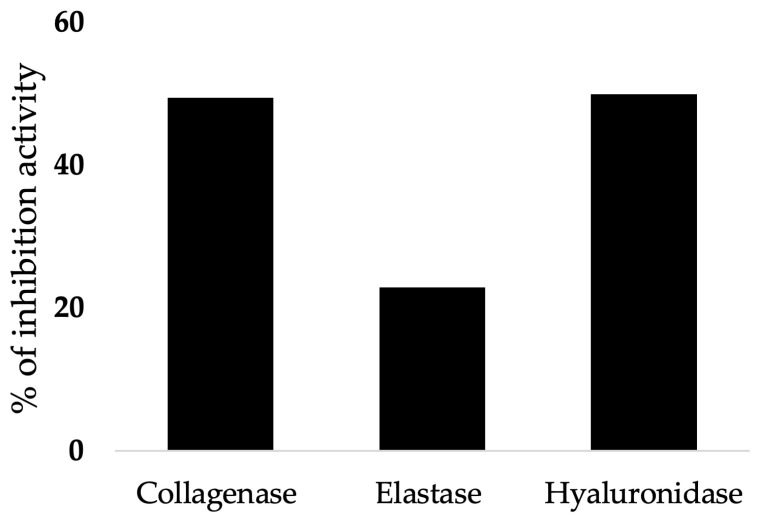
Inhibitory effect of wheat bran extracts on skin aging-related enzymes (collagen, elastin fibers, and hyaluronic acid) “Reprinted (adapted) with permission from [25]. Copyright (2024) American Chemical Society”.

Research has extensively explored the antioxidant properties of wheat bran, but its potential as an inhibitor of these aging-related enzymes remains largely unexplored. As mentioned in the previous paragraphs, some enzymes, such as collagenase, elastase, and hyaluronidase, play crucial roles in the breakdown of extracellular matrix components in the skin, thus contributing to the aging process (Table 1) [53]. The degradation of collagen fibers occurs through two primary phases. In the initial phase (i.e., enzymatic degradation), facilitated by collagenases such as MMP-1, MMP-8, and MMP-13, entire fibers are broken down into diminutive fragments, resulting in the cleavage of the triple-collagen helix into two segments that subsequently undergo spontaneous denaturation to form gelatin [54]. In contrast, the other phase (non-enzymatic degradation), occurs through chemical reactions, such as glycation and oxidation, often exacerbated by environmental factors like UV exposure or metabolic conditions such as diabetes [54]. Elastases can bind to cell surface proteins, creating a microenvironment that promotes elastolysis. This interaction is mediated by exosites distinct from the active site, enhancing the enzyme’s ability to degrade elastin [55]. Hyaluronidase enzymes play a crucial role in the breakdown of hyaluronic acid), a major component of the extracellular matrix. These enzymes facilitate the degradation of hyaluronic acid into smaller fragments, which can have various biological effects. The mechanism of hyaluronidase action involves cleaving the glycosidic bonds within hyaluronic acid, leading to the formation of low-molecular-weight hyaluronic acid fragments [56]. A current study bridges an important gap in the literature and explores the potential of WBEs in anti-aging and skin health applications [25]. The investigation examined the inhibitory impacts of protein hydrolysates and xylo-oligosaccharides derived from wheat bran on the enzymatic activities of collagenase, elastase, and hyaluronidase (Figure 5). The findings indicate that, upon optimized extraction, the wheat bran extract demonstrated significant inhibitory effects on aging-related enzymes, specifically collagenase (49.4%), elastase (22.9%), and hyaluronidase (49.9%) [25]. These enzymes are critical in the degradation of collagen, elastin fibers, and hyaluronic acid, which contributes to the development of sagging, wrinkled, and less elastic skin [13]. The results suggest that WBEs could be valuable ingredients in natural anti-aging and skin health products, offering a multifaceted approach to skin protection and rejuvenation [25]. The results of the multifaceted investigation reflect merit in the future development of novel, natural- based products in both the food and cosmetic industries while also advancing our understanding of the relationships among different bioactive properties in plant extracts. Such findings can contribute to uncovering new potential applications for WBEs. Therefore, future research could focus on identifying and isolating specific compounds responsible for the observed bioactivities, as well as investigating the in vivo efficacy and safety of these extracts. Additionally, further exploration of other potential bioactivities and the development of targeted extraction methods could unlock even more applications for this abundant and sustainable resource.

### 2.5. Other Health Benefits of Wheat Bran

Wheat bran contains essential nutrients, including non-starch polysaccharides like arabinoxylans and β-glucans, which are beneficial for managing cardiovascular diseases, cholesterol, obesity, type-2 diabetes, and cancer [14]. It is a rich source of dietary fiber, which promotes bowel health, reduces cholesterol levels, and lowers the risk of heart disease, diabetes, colon cancer, and obesity [57]. The bran’s mineral content supports metabolic functions and homeostasis, which can be enhanced through processing techniques like hydrothermal treatment and fermentation. Wheat bran is abundant in bioactive compounds such as phenolic compounds and bioactive peptides, which exhibit immunomodulating, antihypertensive, osteoprotective, and antimicrobial properties [57]. Purple wheat bran, in particular, has high levels of anthocyanins, polyphenols, and flavonoids, contributing to its superior antioxidant activity [58]. The incorporation of wheat bran into food products, like bread, cookies, and noodles, is promising because of its health-promoting properties. However, challenges in breadmaking, such as sensory and rheological issues, can be addressed through pretreatments and enzyme additions [57]. Arabinoxylan, a primary component of wheat bran, can be extracted and used in various food applications, enhancing the nutritional profile of these products [59].

## 3. Current Findings

### 3.1. Animal Experiments

To investigate the anti-aging potential of WBEs, we conducted a pilot study using a D-galactose-induced aging model in female Wistar rats. Eight-week-old rats (average weight: 193 g ± 20 g) were obtained from Charles River (O’Fallon, Missouri) and housed in a pathogen-free facility under controlled environmental conditions (12 h light/dark cycle). After a period of acclimation, the rats were randomly assigned to one of the following four groups: Group 1—saline-injected control (i.e., negative control), fed an AIN-93G diet; Group 2—D-galactose-injected positive control, fed an AIN-93G diet; Group 3—D-galactose-injected, fed a 5% WBE-supplemented diet; and Group 4—D-galactose-injected, fed a 10% WBE-supplemented diet. Following six weeks of treatment, blood, skin, and liver samples were collected for the analysis of aging-related biomarkers.

### 3.2. Superoxide Dismutase (SOD) Activity

**Figure 6:**
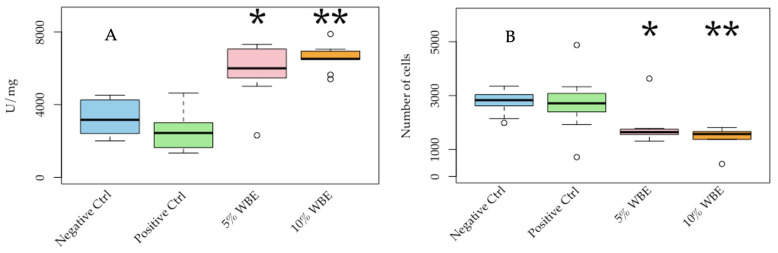
Anti-aging and senescence biomarker analysis in a D-galactose-induced aging rat model. Female Wistar rats were divided into four groups—saline-negative control, D-galactose positive control, and D-galactose positive control with 5% or 10% wheat bran extract diets—for 6 weeks. (**A**) SOD inhibition rate; 1 unit (U) represents the amount of SOD in a 20 µL erythrocyte sample, which shows a 50% inhibition of WST reduction. (**B**) β-galactosidase activity in liver samples. * *p* < 0.05 and ** *p* < 0.001 compared with the saline-negative control. Data represent the mean ± SD (*n* = 10).

As shown in Figure 6A, the means of the erythrocyte SOD activity, measured using a Thermo Multiskan GO Microplate Reader, were as follows: Group 1 (saline-negative control): 3304 U/mg; Group 2 (D-galactose positive control): 2548 U/mg; Group 3 (5% WBE diet): 5842 U/mg; and Group 4 (10% WBE diet): 6565 U/mg. The D-galactose-induced reduction in SOD activity was significantly reversed in both the 5% and 10% WBE-supplemented groups (*p* < 0.0001).

### 3.3. β-Galactosidase Activity

β-Galactosidase (β-Gal), a lysosomal enzyme associated with cellular aging, particularly in the liver, was also measured (Figure 6B). The results show that liver β-GAL activity decreased significantly in both the 5% and 10% WBE-fed groups compared to both negative and positive control , indicating a smaller proportion of senescent cells in the WBE-treated groups. The older neurons exhibited more signs of senescence, further supporting the anti-aging potential of WBEs.

In addition, SOD2 and catalase activities were further measured in both skin and liver samples. However, the results do not show significant differences among the four groups. Therefore, the results are not presented. The significant increases in erythrocyte SOD and significant decreases in liver β-Gal activities in the WBE-fed groups suggest that WBEs enhance antioxidant defenses against oxidative stress, highlighting their potential role in anti-aging interventions. Further research with varying dosages, extended treatment durations, and larger sample sizes may be warranted to fully elucidate the effects of WBEs on aging and to explore their potential to promote healthy aging.

## 4. Application of Wheat Bran Extract

### 4.1. In Food Industries

Wheat bran extract is increasingly recognized for its valuable contributions to the food industry, primarily due to its rich nutritional profile and functional properties. The extract, derived from the outer layers of wheat grain, is a source of dietary fiber, proteins, and bioactive compounds, making it a versatile ingredient in food applications. Wheat bran extract is rich in arabinoxylans, which are the primary hemicelluloses in wheat bran. These compounds are known for their health benefits and functional properties in food products, such as improving texture and stability [59]. The water-soluble extract from wheat bran contains a significant amount of carbohydrates (50.3%), proteins (24.5%), soluble fibers (6.7%), insoluble fiber (2.9%), and ash (13.0%) [60]. The study revealed that adding only 7.5% wheat bran aqueous extract (WBE) could increase bread-specific volume from 4.18 cm^3^/g to 4.84 cm^3^/g. On top of that, dough enriched with WBE demonstrated elevated peak, setback, breakdown, and final viscosities, in addition to enhanced storage and loss modulus. Analysis via scanning electron microscopy additionally indicated that the incorporation of WBE facilitated the interaction of protein and starch components within the dough [61].

A recent study isolated and purified a novel antimicrobial peptide (WBp-1) from wheat bran which exhibited considerable antibacterial potency against *Listeria monocytogenes*, with a minimum inhibitory concentration measured at 150 μg/mL [62]. Moreover, a soluble extract of wheat bran at 0.5% exhibited anti-biofilm properties, preventing and disrupting biofilm formation. Additionally, the wheat bran demonstrated an ability to modulate bacterial quorum-sensing mechanisms, evidenced by its lactonase activity that decreased acyl-homoserine lactones levels in the medium [63]. Another investigation employed both 0.4% and 0.8% methanol/water wheat bran extract (MWBE) in the formulation of hamburgers [61]. Around a 25% suppression of Enterobacterial proliferation was observed by MWBE compared to the control sample [61]. Regarding the coloration of the hamburgers, at the end of the storage period, it was observed that the hamburgers containing MWBE exhibited a color retention of 91.93–80.77%, whereas the control sample demonstrated a color retention of 98.85–93.98%, indicating that the incorporation of these additives contributes to superior color preservation. Furthermore, the MWBE exhibited an enhanced antioxidant capacity, demonstrating an increase of up to 20% relative to the control sample [61]. Meatballs with 20% wheat bran exhibited elevated ash and protein levels, enhanced lightness and yellowness, and diminished moisture, salt, and weight loss, alongside reduced redness. Sensory analysis indicated a significant variance among meatball samples, with control samples demonstrating superior acceptability [64]. A study examined the antimicrobial and antioxidant properties of phytic acid and phytate from wheat bran [65]. Gram-positive bacteria exhibited higher sensitivity to phytic acid compared to Gram-negative bacteria. *Bacillus anthrakoid* and *Staphylococcus aureus* demonstrated increased sensitivity at 5% and 6% concentrations of pure phytate [65]. The inhibition zones for *Escherichia coli* O157:H7 and *Staphylococcus aureus* were 20.33 mm and 38.67 mm, respectively, at 6% pure phytate. Furthermore, the aqueous extract of wheat bran achieved a free radical scavenging activity of 36.16% [65]. The researchers concluded that wheat-bran-extracted phytate possesses significant antimicrobial and antioxidant properties, suggesting its potential as a natural preservative. Overall, the WBEs hold significant potential for the food industry due to their rich composition of bioactive compounds and dietary fibers, as well as their ability to be transformed into functional food ingredients. These extracts can be utilized in various applications, ranging from enhancing the nutritional profile of food products to serving as substrates in biotechnological processes.

### 4.2. In Cosmetics Industries

WBEs could be a potential ingredient source in the cosmetics industry because of their functional properties, including emulsion stability, skin-whitening effects, and antimicrobial capabilities. WBEs could be utilized in some cosmetic formulations, offering natural and effective alternatives to synthetic ingredients. Wheat-bran-derived lipids have been found to enhance the stability of emulsions, and emulsions from natural sources are in high demand in cosmetic formulations [66]. The emulsions with wheat-bran-derived lipids demonstrated higher emulsion stability (49.17–52.33%) compared with the control emulsion made with soybean oil (46.00%). This indicates that the addition of wheat-bran lipids enhances the stability of an emulsion without causing particle separation. This study found that the skin-whitening effect of the emulsions increased with the concentration of wheat-bran-derived lipids [24]. These findings indicate the potential application of wheat-bran-derived lipids in the cosmetics sector.

Wheat bran extract may enhance skin hydration, softness, and smoothness. These extracts are particularly beneficial in skincare formulations, as well as in shampoos and scalp treatments. Previous research indicates that the combination of wheat bran extract with carboxylic acid alkyl ester and diol enhances combability, shine, elasticity, volume, and shape retention and minimizes damage [67]. Moreover, ferulic acid encompasses a multitude of prospective therapeutic applications, functioning as a scavenger of free radicals and serving as a protective agent against ultraviolet radiation-induced skin damage. Research has determined that there exists an amount of up to 5157 ± 66 mg of alkali-extractable ferulic acid per kilogram of dry destarched wheat bran [68]. Phycocyanobilin exhibits potential benefits for human skin, including wrinkle reduction and anti-inflammatory properties. Research indicates that phycocyanobilin extract from marine Arthrospira maxima possesses skin anti-wrinkling effects linked to antioxidant activity and decreased intracellular reactive oxygen species. Furthermore, phycocyanobilin extracts significantly enhance collagen synthesis while reducing matrix metalloproteinases (MMP-1) levels. Notably, the anti-wrinkling effects of phycocyanobilin extracts are markedly amplified by incorporating WBEs, achieving up to 20–30% enhancement across different concentrations, likely due to the synergistic interaction of soluble globulins and other bioactive compounds in WBEs [69].

WBEs, particularly xylo-oligosaccharides (XOS) and protein hydrolysates, have demonstrated strong antioxidant capacities. These extracts inhibit enzymes such as collagenase, elastase, and hyaluronidase, which are associated with skin aging. The inhibition of these enzymes suggests potential applications in cosmetic products aimed at reducing skin wrinkles and maintaining skin elasticity [25]. The phenolic content in WBEs, especially from hard wheat bran, has been shown to significantly inhibit hyaluronidase activity, reaching up to 49.9% inhibition at a concentration of 0.5 mg/mL. This highlights the potential of WBEs in developing functional ingredients for anti-aging purposes [25]. Wheat bran extract has also been studied for its neuroprotective effects, particularly against β-amyloid-induced cell death, which is a hallmark of Alzheimer’s disease. The extract was found to protect neuronal cells from apoptosis and improve memory impairment in animal models, suggesting its potential in managing neurodegenerative conditions associated with aging [70].

## 5. Factors That Affect the Yield of WBEs

The extraction of wheat bran presents several challenges and limitations, primarily due to its complex structure and the need for efficient, sustainable methods. Several extraction methods including conventional solvent extraction, ultrasound-assisted extraction, and microwave-assisted extraction have been employed to isolate useful compounds from wheat bran [57]. Wheat bran extraction methods possess effects on biological activities, including antioxidant, anti-inflammatory, and antimicrobial properties [71]. For instance, enzymatic hydrolysis of wheat bran to obtain XOS and protein hydrolysates has shown promising antioxidant capacities [72]. Methanol extracts from colored wheat bran, such as ‘Ariheuk’, exhibit strong antioxidant activities due to a high anthocyanin content [5]. While the extraction method significantly affects the biological activities of wheat bran, the choice of method should align with the desired application, whether it be for antioxidant, anti-inflammatory, or antimicrobial purposes. The presence of specific compounds like anthocyanins and phytic acid plays a crucial role in determining the efficacy of these extracts. In addition to the above, some researchers have also investigated the potential of WBEs in food preservation and as natural additives in functional foods [30]. For instance, arabinoxylans are the primary hemicellulose in wheat bran, known for their ability to form gels and films, which can be used in food packaging and as delivery systems for food ingredients [59]. Wheat bran contains aqueous-phase soluble proteins that can stabilize food systems, particularly in liquid and semi-solid foods. These proteins are extracted through physical and biochemical modifications to overcome barriers such as intact cell walls [73]. Supercritical CO_2_ extraction of wheat bran yields extracts rich in phenolic compounds, which exhibit significant antioxidant activity. This property is crucial for preventing oxidative spoilage in foods [74]. Biochemical conversion processes, such as the Maillard reaction and enzymolysis, improve the flavor profile of wheat bran, making it a more acceptable food ingredient. This enhancement is particularly beneficial in products like steamed buns, where flavor and sensory attributes are critical [3].

## 6. Potential Risks and Mitigations

Wheat bran extracts, though widely recognized for their nutritional and bioactive properties, come with specific safety concerns that warrant consideration. Individuals with wheat allergies or celiac disease may experience adverse reactions due to the presence of wheat proteins or gluten contamination, even in trace amounts. The high phytic acid content in wheat bran can act as an antinutrient by binding essential minerals such as calcium, iron, and zinc, reducing their bioavailability and potentially leading to deficiencies with excessive intake. Additionally, overconsumption of wheat bran fiber can cause digestive issues, including bloating, gas, or diarrhea, particularly in individuals unaccustomed to high-fiber diets. Furthermore, the high fiber content and bioactive compounds in wheat bran can interfere with the absorption of medications, reducing their effectiveness. Long-term toxicological data on concentrated or bioactive-enriched wheat bran extracts remain limited, necessitating further research to ensure safety. To mitigate these risks, quality control measures, proper dosage recommendations, and consumer awareness about potential interactions and allergens are essential for the safe use of wheat bran extracts.

## 7. Challenges and Future Perspectives

There is a critical need for comprehensive in vivo studies to fully understand the efficacy and safety of WBEs. Future research should focus on bioavailability studies to determine how effectively the bioactive compounds in WBEs are absorbed, metabolized, and distributed in the body. Additionally, factors such as stability during food processing, storage, and formulation into cosmetic products must be thoroughly explored to ensure that the active compounds retain their potency. Animal studies can provide insight into the systemic effects of WBEs, including their antioxidant and anti-inflammatory effects across various organs and tissues. For cosmetic applications, dermal absorption studies and clinical trials are essential to evaluate the efficacy of WBEs in improving skin health and reducing signs of aging, particularly in real-world conditions. Furthermore, understanding the mechanisms of action at the molecular level could guide the development of more targeted applications. Long-term safety studies are crucial to assess any potential adverse effects, allergenic responses, or interactions with other substances in both food and cosmetic matrices. In addition to efficacy, these studies are necessary for securing regulatory approvals and boosting consumer confidence. Challenges also remain in scaling up extraction and production processes while ensuring cost-effectiveness and sustainability. Overcoming these barriers will unlock the full potential of WBEs as bioactive ingredients in anti-aging formulations for both food and cosmetic applications.

## Figures and Tables

**Table 1:** Summary of skin-related enzymes and their role in skin aging.

Enzyme	Target	Role in Skin	Effect on Aging	Potential Benefits of Inhibition
Collagenase	Collagen	Breaks down collagen fibers	This leads to loss of skin firmness and the formation of wrinkles.	Maintenance of skin structure and elasticity.
Elastase	Elastin	Degrades elastin fibers	Results in decreased skin elasticity and sagging.	Preservation of skin elasticity and bounce.
Hyaluronidase	Hyaluronic acid	Breaks down hyaluronic acid	Reduces skin hydration and volume.	Retention of skin moisture and plumpness.

## Data Availability

No new data were created or analyzed in this study. Data sharing is not applicable to this article.
